# Supplementary material for: Protein Profiling of Placental Extracellular Vesicles in Gestational Diabetes Mellitus
Source: Int J Mol Sci. 2024 Feb 6;25(4):1947. doi: 10.3390/ijms25041947 (PMC10887986; doi:10.3390/ijms25041947)

Supplementary Figures:

Table S1: Primary Antibodies for EV characterisation

**Antibody to**	**Alix**	**PLAP**	**Syntenin**	**CD9**	**CD63**	**Cyt-C**
Full Name	ALG-2-Interacting Protein X	Placental Alkaline Phosphatase	Syntenin	CD9	CD63	Cytochrome-C
Application	WB	WB	WB	WB	WB	WB
Host Species	Mouse	Mouse	Rabbit	Mouse	Mouse	Mouse
Antibody Isotype	IgG	IgG	IgG	IgG1	IgG1k	IgG2bk
Dilution	1:100	1:1000	1:1000	1:1000	1:1000	1:500
Company	Santa Cruz Biotechnology	In-house antibody	Abcam	Santa Cruz Biotechnology	Santa Cruz Biotechnology	Santa Cruz Biotechnology
Catalogue number	Sc-53538	NDOG2	Ab133267 data	Sc-59140 data	Sc-59286 data	Sc-13156

Table S2: Secondary antibodies for EV characterisation

**Antibody**	**Horse Anti-Mouse**	**Goat Anti-Rabbit**
Application	WB	WB
Conjugation	Horseradish peroxidase	Horseradish peroxidase
Antibody Isotype	IgG	IgG
Dilution	1:1000	1:1000
Company	Cell Signaling Technology	Cell Signaling Technology
Catalogue number	7076S	7074S

Figure S1: Original Western blots (upper panel) and Ponceau Red loading control (lower panel) for EV characterisation

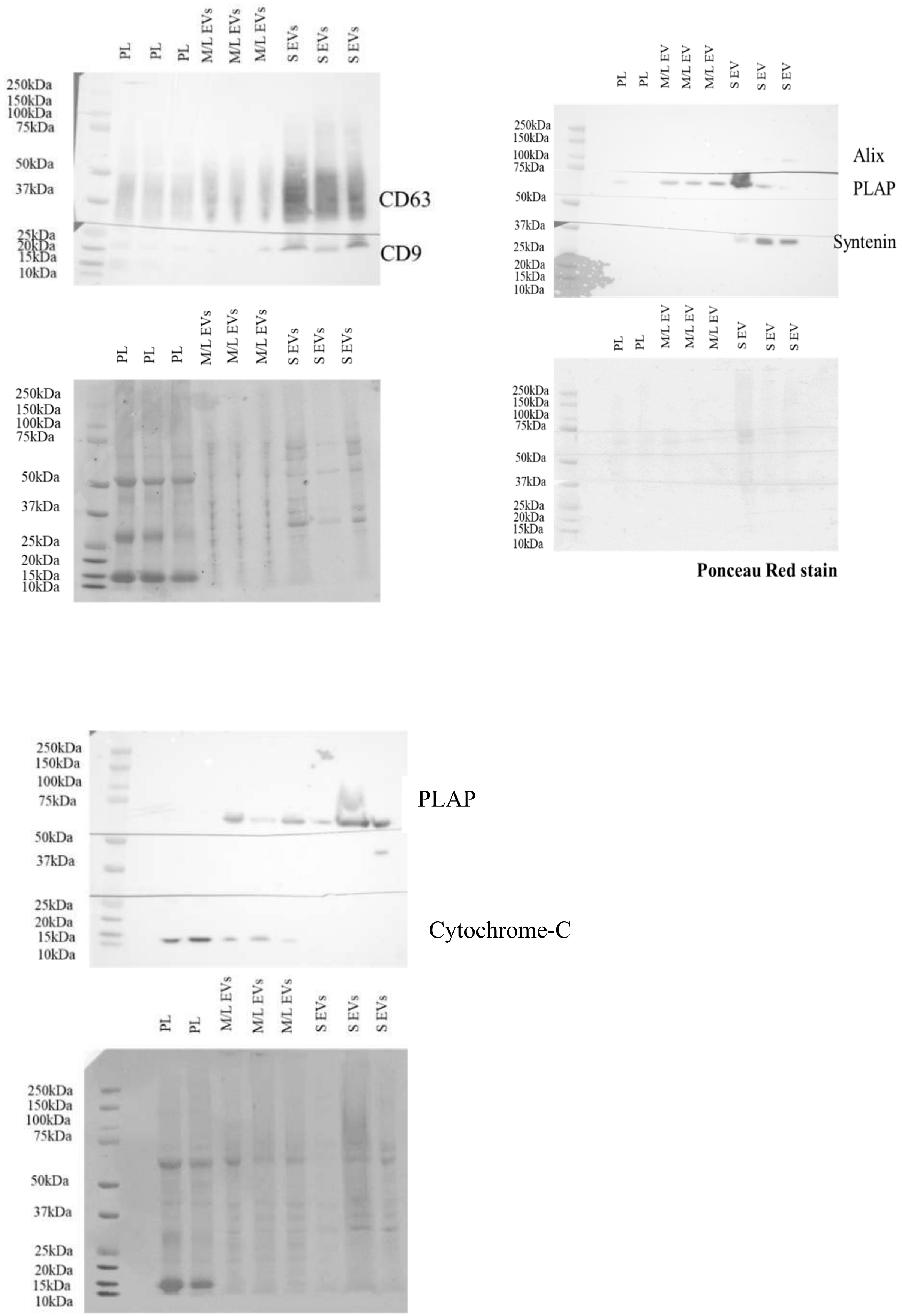

Supplement: Supplementary file 1 [file ijms-25-01947-s001.zip › ijms-2829137-supplementary.pdf]
